# Immunohistochemical expression of hormone receptors, Ki-67, endoglin (CD105), claudins 3 and 4, MMP-2 and -9 in endometrial polyps and endometrial cancer type I

**DOI:** 10.2147/OTT.S160014

**Published:** 2018-07-09

**Authors:** Gustavo Filipov Peres, Daniel Spadoto-Dias, Flávia Neves Bueloni-Dias, Nilton José Leite, Leonardo Vieira Elias, Maria Aparecida Custódio Domingues, Carlos Roberto Padovani, Rogério Dias

**Affiliations:** 1 Department of Gynecology and Obstetrics, ddias.sp@fmb.unesp.br; 2 Department of Clinical Pathology; 3 Department of Biostatistics, Institute of Biosciences, Botucatu Medical School, São Paulo State University, Botucatu, São Paulo, Brazil

**Keywords:** endometrium/pathology, immunohistochemistry, endometrial neoplasia, polyps/epidemiology

## Abstract

**Objective:**

The aim of this study was to investigate the malignant potential of endometrial polyps (EP) by assessing the immunoexpressions of both estrogen receptor (ER) and progesterone receptor (PR), Ki-67 cell proliferation index, neovascularization network (endoglin – CD105), cellular adhesion molecules (claudins 3 and 4), and extracellular matrix proteins (MMP-2 and -9) in both EP and endometrioid adenocarcinoma (type I) in comparison with the normal endometrium.

**Study design:**

This is a cross-sectional comparative study. Patients were identified from the database of Botucatu Medical School, São Paulo State University (BMS-UNESP) Clinical Pathology Laboratory.

**Setting:**

The study was conducted using a convenience sample of patients attending the Sectors of Gynecologic Endoscopy and Family Planning and Gynecologic Oncology of the Department of Gynecology and Obstetrics of BMS-UNESP, Brazil.

**Patients:**

A total of 90 women were allocated into the following three groups: EP without atypia (EP, n=30), endometrioid endometrial cancer (EC, n=30), and normal endometrium (control, n=30).

**Methods:**

Epidemiological and clinical data were obtained by reviewing medical records. Adenocarcinoma and control cases were assessed using the tissue microarray technique. The immunoexpressions of ER, PR, Ki-67, CD105, claudins 3 and 4, and MMP-2 and -9 were assessed in paraffin blocks containing sections of the largest polyploid lesion fragment and tissue microarray recipient blocks.

**Major results:**

Compared to the control group, significant differences in the expression of ER (*P*<0.001), PR (*P*<0.05), Ki-67 (*P*<0.001), CD105 (*P*<0.001), and claudin 3 (*P*<0.001) were observed in EP and EC. No significant differences were found between EP and EC (*P*≥0.05). MMP-2 and -9 expression were nearly absent in all groups.

**Conclusion:**

The malignant potential of EP could not be determined through the immunohistochemical parameters used in this study. No MMP-2 or -9 expression was observed in any endometrial tissue sample. Further studies are necessary for a better understanding of the biomolecular mechanisms underlying endometrial carcinogenesis.

## Introduction

Endometrial polyps (EP) are often asymptomatic and detected incidentally during routine pelvic transvaginal ultrasonography.^1^ They can be associated with abnormal uterine bleeding and are estimated to be responsible for up to 39% of cases of irregular bleeding during menacme and 21%–28% of cases of postmenopausal bleeding.^2^,^3^ In the general population, the prevalence of EP is reported to range from 25% to 30%, with an incidence peak in women aged 40–60 years. After menopause, EP prevalence is twofold higher (11.8%) than during menacme (5.8%).^4^–^6^ The preponderance of polyps at perimenopause and in the first year after menopause, added to the inconvenience represented by potential bleeding episodes, indicates that EP is an important condition in the differential diagnosis of endometrial cancer (EC).^2^,^3^

Endometrial adenocarcinoma is the sixth most common type of cancer among women, with 290,000 new cases/year worldwide.^7^ In the USA, EC was estimated to be diagnosed in 52,630 women in 2014, with 8,590 succumbing to their disease.^8^ According to the data collected in the European Union, there were 55,941 cases followed by 12.903 deaths related to the disease in 2008, with an incidence rate of 16.2/100,000.^9^ In Brazil, the incidence of endometrial adenocarcinoma is ~5.8 cases/100,000 women, with a higher prevalence between the age of 50 and 65 years.^10^ The incidence of EC in women with EP is ~3.5% (range 0%–4.8%) with an increased risk in women with postmenopausal bleeding.^2^,^11^ Nonetheless, the rates of hyperplasia and polyp malignancy may be similar among women with bleeding (3.2%) and without bleeding (3.9%), depending on the population studied.^12^

Aiming at predicting EP malignant potential, immunohistochemical studies using several biomarkers have been conducted to elucidate the molecular mechanisms underlying endometrial carcinogenesis.^13^–^15^ Histochemical studies on the expression of hormone receptors, oncogenes, and tumor suppressor genes; antiapoptotic proteins related to mitotic activity and neoangiogenesis; and intercellular adhesion molecules and extracellular matrix proteins have yielded conflicting results regarding their role in the development of endometrial neoplasia.^11^,^16^–^20^

The objective of this study was to investigate EP malignant potential by assessing the immunoexpression of the estrogen receptor (ER), progesterone receptor (PR), cell proliferation index (Ki-67), neovascularization network (endoglin – CD105), cellular adhesion molecules (claudins 3 and 4), and extracellular matrix proteins (metalloproteinase-2 [MMP-2] and metalloproteinase-9 [MMP-9]) in both EP and endometrioid adenocarcinoma (type I) in comparison with the normal endometrium to better understand the mechanism of malignant degeneration associated with EP and to establish criteria for the clinical and surgical management of this condition.

## Patients and methods

This cross-sectional comparative study was conducted using a convenience sample of patients attending the Sectors of Gynecologic Endoscopy and Family Planning and Gynecologic Oncology of the Department of Gynecology and Obstetrics of Botucatu Medical School, São Paulo State University (BMS-UNESP), Brazil. Patients were identified from the database of BMS-UNESP Clinical Pathology Laboratory. Epidemiological and clinical data were obtained by reviewing medical records after approval by the institutional Committee of Research Ethics from Botucatu Medical School process number 272.637, and written informed consent was obtained from all patients.

The study population consisted of 90 women who were allocated into the following three groups: EP, EC, and normal endometrium (control). EP included 30 women who underwent surgical hysteroscopic polypectomy and were diagnosed with EP without atypia histopathologically. The selection of only EP without atypia was limited by the difficulty in finding isolated atypia in EP. EC consisted of 30 women who underwent total hysterectomy and bilateral adnexectomy with pelvic lymph node sampling due to endometrioid adenocarcinoma (according to the World Health Organization Classification System) (OMS 2003). The control group included 30 women with no history of hormone replacement therapy (HRT), paired by age and reproductive stage to the EC group, who underwent uterine prolapse surgery and were histopathologically diagnosed with a normal endometrium (control group).

Adenocarcinoma and control cases were assessed using the tissue microarray (TMA) technique validated for EC (Figure S1).^21^ Having identified the regions of interest in H&E-stained slides, round cores (1 mm diameter) were punched out the corresponding paraffin block and placed in a recipient block using a TMA device (Beecher Instruments Inc, Silver Spring, MD, USA). The spacing between cores was 0.2 mm. Each case sample was then mapped to a grid. TMA was not used for the analysis of EP cases because tissue fragments were too small. Paraffin blocks containing sections of the largest polyploid lesion fragment were selected for immunohistochemical assessment. TMA recipient blocks and original EP blocks were cut into 3-µm-thick sections, mounted on silanized slides, deparaffinized at ~65°C for ~1 hour, and buffered with a retrieval solution (Target Retrieval Solution; Dako, Santa Clara, CA, USA) for 20 minutes in an automated manner (PT Link; Dako).

Sections were incubated with primary antibodies in an automated system (Autostainer Link 48; Dako) for 20–30 minutes (depending on the antibody) at the following dilutions: ER (FLEX Monoclonal Mo a Hu Estrogen Receptor, Clone 1D5, RTU; Dako) at 1:100; PR (FLEX Monoclonal Mouse, X-Hu Progest Recept, Clone PgR 636, RTU; Dako) at 1:200; CD105 (Monoclonal Mouse Anti-Human CD105, Endoglin Clone SN6h, ready to use; Dako); claudin 3 (Rabbit polyclonal Claudin 3 antibody; Dako) at 1:100; claudin 4 (Mouse monoclonal Claudin 4 antibody Clone 3E2C1; Dako) at 1:50; metalloproteinase 2 (Mouse Anti-MMP2 Monoclonal Antibody, Clone CA-4001/CA719E3C; Abcam, Cambridge, MA, USA) at 1:50; and metalloproteinase 9 (Mouse Anti-MMP9 Monoclonal Antibody, Clone 15W2; Abcam) at 1:100. In the same system, sections were also incubated with secondary antibodies (EnVision FLEX; Dako) for 20 minutes. 3,3-Diaminobenzidine (DAB) was used as chromogen (10 minutes), and sections were counterstained with Harris Hematoxylin (5 minutes).

The percentage of ER and PR positively stained was estimated semiquantitatively, as previously described.^22^–^24^ Using 200× magnification throughout the entire tissue section, hormone receptor expression was classified, based on the number of nuclei stained and staining intensity as either negative (absent or weak staining in <10% of the cell nuclei) or positive, which was graded as 1+ (up to 25%), 2+ (26%–50%), 3+ (51%–75%), and 4+ (76%–100%). The glandular epithelium was the only compartment scored; positivity in the stromal compartment was not taken into account (Figure S2).

Microvessel density was determined by endoglin (CD105) immunostaining. Three fields of the highest vascular density were identified under 40× magnification. The number of vessels in each field was counted under 200× magnification, and microvessel density was estimated as the mean number of vessels found in the three fields. In TMA cores, the number of microvessels per core was counted under 200× magnification (Figure S3). A vessel lumen was not required for the identification of microvessels – any stained cell with endothelial characteristics was counted as a microvessel.^11^,^25^ Cell proliferative index was determined according to the percentage of Ki-67 labeled nuclei per hotspot in conventional slides or in 100 cells/core in TMA slides, under 200× magnification (Figure S4).

The expression of claudins 3 and 4 was assessed under 200× magnification using a score system developed by our team in a previous study (Ângela Favorito Santarém Tonon, unpublished data, 2013), which is based on the pattern of membrane staining (0=negative, 1=focal, 2=diffuse, and 3=diffuse membranous and cytoplasmic staining), staining intensity (0=negative, 1=weak, 2=moderate, and 3=strong), and the percentage of stained cells (0=0%–5%, 1=6%–30%, 2=31%–50%, 3=51%–80%, and 4=81%–100%) in each EP slide or TMA core (Figure S5). MMPs’ expression in the cytoplasm of epithelial cells was scored according to staining intensity (0=negative, 1=weak, 2=moderate, and 3=strong) and percentage of stained cells (0=0%–5%, 1=6%–30%, 2=31%–50%, 3=51%–80%, and 4=81%–100%) (Figure S6). Appropriate positive and negative controls were used for each biomarker.

For data analysis, measures of location and variability were calculated. Mean, SD, median, and minimum/maximum values for quantitative variables and absolute frequency and percentage for qualitative variables were estimated. Qualitative variables were analyzed using the test of Goodman for contrasts among and within multinomial populations.^26^,^27^ Normally distributed quantitative variables were compared using one-factor analysis of variance followed by Tukey’s test for multiple comparisons (factor for three groups) and Student’s *t*-test for independent samples (factor for two groups). Non-normally distributed quantitative variables were assessed using the Kruskal–Wallis nonparametric test with Dunn’s test (three levels) and Mann–Whitney test (two levels) for multiple comparisons.^28^ Data analysis was performed using SPSS for Windows, V. 21.0 with significance level set at 5%.

## Results

Table 1 shows the clinical, epidemiological, and anthropometric characteristics of the study participants with EP (n=30) or type I EC (n=30). Study groups were homogeneous with respect to parity, body mass index (BMI), use of HRT, arterial hypertension, diabetes mellitus, and breast cancer (*P*≥0.05). Significant differences were observed in patient age and reproductive life stage. Age was more advanced (mean 60.3 vs 52.2 years; *P*<0.001) and a postmenopausal status was more frequent (90 vs 56.7%; *P*<0.05) in women with cancer than in women with EP (Table 1).

Table 2 shows the immunohistochemical profile of the 90 study participants according to group (EP, EC, and normal endometrium – control). Endometrial neoplasia, diagnosed as endometrioid adenocarcinoma, was graded and staged, according to the International Federation of Gynecology (FIGO 2009), as follows: grade I=25, grade II=3, and grade III=2 and stage IA=21, stage IB=4, stage II=2, stage IIIA=2, and stage IV=1. The mean age in the control group (paired by age and reproductive stage to the EC group) was 64.1 (±9.2 years) (*P*≥0.05).

Significant differences were observed among groups regarding hormone receptors’ immunoexpression, cell proliferation index, neovascularization marker, and cell adhesion molecules (Table 2). ER expression was higher in the control group (4.0+ [0.0;4.0+]) than in EP (2.5+ [1.0+;4.0+]) and EC (1.0+ [0.0+;4.0+]) (*P*<0.001). PR expression was higher in the control group (4.0+ [0.0;4.0+]) than in EC (2.0+ [0.0+;4.0+]) (*P*<0.05). The expression of endoglin (CD105) and Ki-67 was higher in EP (20.8 [12.0;48.7] and <1.0% [0.0;15.0%], respectively) and EC (18.0 [0.0;45.0] and 2.0% [0.0;100.0%], respectively) than in controls (6.5 [0.0;26.0] and 0.0% [0.0;0.0], respectively) (*P*<0.001).

Claudin 3 showed predominantly diffuse membrane staining in endometrial endometrioid cancer (2.0 [0.0;3.0]) in contrast to a focal membrane staining pattern in the control group (1.0 [0.0;1.0]) (*P*<0.005). Claudin 3 staining was stronger in both EP (3.0 [0.0;3.0]) and EC (3.0 [0.0;3.0]) than in the normal endometrium (2.0 [0.0;3.0]) (*P*<0.001). Claudin 3 count was greater in normal endometrium tissue (4.0 [0.0;4.0]) than in EP (3.0 [0.0;4.0]) (*P*<0.05) samples. With relation to claudin 4 and metalloproteinases (MMP-2 and -9), no significant difference was observed among groups (*P*≥0.05) (Table 2).

## Discussion

The pathogenesis of EP is still not completely understood, and there is still debate regarding the management of this condition, particularly in postmenopausal asymptomatic women.^2^,^3^,^29^ The risk factors for malignancy in EP are the same as those reported for EC, namely advanced age, nulliparity, early menarche, late menopause, obesity, hypertension, diabetes, and use of tamoxifen.^3^,^6^,^30^

In this study, hypertension and obesity rates were high, but no differences were observed between women with EP and those with EC of the endometrioid type I. In women with EC, however, age was higher and time since menopause was longer, which might have contributed to increase the risk of malignancy. Although hypertension, diabetes, and obesity are associated with EP, studies have shown that their influence loses statistical significance on multivariable logistic analysis, indicating that age is the only independent risk factor.^31^,^32^

Steroid hormones, especially estrogen, play a key role in the pathogenesis of endometrial hyperplasia and endometrioid adenocarcinoma type I.^8^ Their lipophilic structure allows estrogen to pass freely through the cell membrane and bind to the ER in the nucleus, controlling the expression of several genes, including those that encode PR and growth factors.^19^ Exposure to estrogen unopposed by progesterone promotes the activation of oncogenes and the inactivation of tumor suppressor genes, impairing cell cycle regulation and leading to dysfunction of the proteins involved in tumor invasion and progression.^19^,^33^ An imbalance between cell proliferation and apoptosis, influenced by the concentration of sex steroids, is thought to be one of the mechanisms underlying the development of endometrial disorders, either benign or malignant.^15^,^34^,^35^ Thus, determining endometrial hormone response in the normal endometrium, as well as in benign, premalignant, and malignant lesions is very important for the characterization of potentially malignant changes.

Previous studies have demonstrated higher estrogen and progesterone expression in the epithelium of EP in comparison with normal endometrial tissue. These findings suggest that EP have a higher sensitivity to estrogen and progesterone and may develop even in the presence of low serum hormone concentrations.^19^,^23^,^24^,^36^ In this study, however, although EP and EC were associated with hormone hyperstimulation, ER concentration was significantly higher in women with a normal endometrium than in those with EP or EC (4.0+ [0.0;4.0+]^b^ vs 2.5+ [1.0+;4.0+]^a^ vs 1.0+ [0.0;4.0+]^a^, respectively; *P*<0.001). PR concentration, in turn, was higher in the normal endometrium than in EC (4.0+ [0.0;4.0+]^b^ vs 2.0+ [0.0;4.0+]^a^, respectively; *P*<0.05). A positive feedback mechanism in response to the low postmenopausal levels of endogenous hormones might explain the increased number of receptors seen in the control group.^37^ Notably, in agreement with a recently published report, our results show that both ER and PR expression were higher, though not statistically significant, in EP than in EC (Table 2).^11^ Nondifferentiation of the neoplastic tissue, which occurs as the disease progresses, associated with the loss of its regulatory mechanisms, might be responsible for this finding.^38^,^39^ Likewise, PR decrease may be associated with abnormal decidualization that can contribute to the development of endometrial lesions.^19^ Some studies suggest that EP correspond to an earlier stage where the tissue is well differentiated and responds to hormone production similarly to the adjacent endometrium. Endometrial neoplasia, in turn, is a more advanced stage of the hyperplastic process where the endometrium has lost its ability for hormone response and thus becomes disorganized, causing invasion and dissemination of the disease.^38^,^40^ Deter mining the type of the polyp and accurately distinguishing the presence of atypia from its absence, which is related to increase or decrease in the number of hormone receptors, might explain the conflicting data found here. However, further investigation is necessary.

The number of newly formed vessels, estimated using endoglin (CD105) in this study, significantly differed among the normal endometrium, EP, and EC (6.5 [0.0;26.0]^a^ vs 20.8 [12.0;48.7]^b^ vs 18.0 [0.0;45.0]^b^, respectively; *P*<0.001), supporting the view that a neovascularization mechanism underlies the hyperplastic and neoplastic processes. Vascular microdensity via CD105 was higher in complex hyperplasia with atypia when compared with simple hyperplasia.^41^ However, there was no significant difference between EP and EC (Table 2). This is in agreement with recent research demonstrating that using endothelial markers does not allow distinguishing EP from EC.^11^,^42^ Given that both conditions are associated with hyperplasia of the endometrial layer, the vascularization mechanism underlying the hyperplastic process might be the same. However, there is evidence that endoglin (CD105) plays a broader role in modulating the proliferation, adhesion, and migration of neoplastic cells.^40^ It has been suggested that endoglin, at early stages, regulates carcinogenesis suppressing invasion and metastasis, whereas at more advanced stages, its expression increases promoting progression of the disease as well as tumor cell migration and invasiveness, enabling neovascularization.^40^,^43^

In the control group of this study, mitotic activity was absent consistently with an inactive endometrium (0.0% [0.0;0.0]^a^; *P*<0.001). In the groups with EP and EC, there was discrete cell proliferative activity (<1.0% [0.0;15.0%]^b^ vs 2.0% [0.0;100.0%]^b^, respectively; *P*≥0.05). The Ki-67 protein is present during the active phases of the cell cycle (G1, S, G2, and mitosis) but is absent from resting cells (G0). It is an excellent marker for quantifying cell growth in a given cell population.^25^ In neoplastic processes, the index of cell proliferation is expected to be substantially increased, particularly in high-grade tumors, indicating the intense mitotic activity associated with this condition.^20^,^37^ Nonetheless, similar to that estimated using the endothelial marker CD105, Ki-67 proliferation index did not significantly differ between EP and EC in this study. This might be due to the fact that well-differentiated endometrial neoplasia (histological grade I) at an early stage of the disease (FIGO grade I) predominated among the study participants. In this case, tumor proliferative activity in the EC group might not have reached its peak, being slightly increased in relation to that in the EP group. Similarly, the low expression of endoglin found in the EC group might be associated with the mechanism of endoglin regulation seen early in the tumoral process. At later stages, increase in vascular microdensity (CD105) correlates with angiolymphatic invasion and lymph node metastases, indicating poor prognosis.^41^,^44^

Claudins 3 and 4 are proteins found in tight junctions between epithelial cells that act as a paracellular barrier and control the movement of molecules through the intercellular space.^45^ They also take part in the maintenance of cell polarity and in the regulation of cell proliferation and differentiation as they have the capacity to recruit signaling proteins.^45^,^46^ Loss of epithelial integrity with change in claudins’ expression and consequent increased paracellular leakage is believed to play a role in providing a space for tumor cell mobility and increased nutrients’ supply for tumor cells.^45^,^47^ Preliminary studies show that the increased expression of claudins 3 and 4 in EC of serous papillary or clear-cell histology are associated with high rates of recurrence and poor prognosis.^48^

Pan et al,^16^ investigating the role of claudins in EC, found that claudin 3 expression and claudin 4 expression were significantly higher in atypical hyperplasia and in endometrioid adenocarcinoma in comparison with the normal endometrium. In this study, claudin 3 showed predominantly diffuse membrane staining in EC in contrast to a focal membrane staining pattern in the control group (2.0 [0.0;3.0]^b^ vs 1.0 [0.0;1.0]^a^, respectively; *P*<0.005), suggesting that claudin 3 concentration is increased in tumor cell membranes compared to the normal endometrium. Additionally, staining was stronger in both EP and EC samples in comparison to those of the normal endometrium (3.0 [0.0;3.0]^b^ vs 3.0 [0.0;3.0]^b^ vs 2.0 [0.0;3.0]^a^, respectively; *P*<0.001). Staining distribution was more homogeneous in the normal endometrium than in EP (4.0 [0.0;4.0]^b^ vs 3.0 [0.0;4.0]^a^, respectively; *P*<0.05). These findings suggest an increased claudin 3 concentration in both EP hyperplastic tissue and EC neoplastic tissue associated with an irregular distribution, compatible with a disorganized rearrangement of these proteins, which is expected in tissue dysplasias.

In relation to claudin 4, differences were observed only in its tissue distribution (Table 2) that, although not significant, suggest dysfunctional cell activity in dysplastic tissue through an abnormal biomolecular behavior characterized by the irregular hyper-uptake of this protein in the tissues analyzed.

MMPs are zinc (Zn^2+^)-dependent endopeptidases thought to play a major role in tumor invasion and metastasis, which require the proteolysis of basal membranes and extracellular matrix.^49^ MMPs have been associated with the aggressive behavior of some neoplasias, and the immunoexpression of MMP-2 and -9 seems to be of prognostic value in endometrial carcinoma.^17^,^18^ Recent studies suggest that MMPs are involved in several other processes associated with tumor development, as they regulate tumor growth and apoptosis, and promote angiogenesis, loss of cell adhesion, invasion, and metastasis.^49^ Erdemoglu et al^49^ demonstrated that MMP-2 and -9 are expressed in both pre- and postmenopausal polyps and that their expression may vary with hormonal status.

In this study, MMP-2 and -9 expression were not observed in any of the groups. This indicates that endometrioid endometrial neoplasia development possibly occurs through an independent pathway or change in the production of extracellular matrix proteins only at later stages of the process. Further investigation is needed to elucidate the role of MMP-2 and -9 in the pathogenesis of endometrial hyperplasia and neoplasia.

The major limitations of this study include the absence of samples of EP with atypia and the fact that this investigation was limited to cases of endometrioid (type I) EC, well-differentiated tumors very similar to the normal endometrium and polyps that might partially explain the results obtained. Moreover, the selection process was limited by the difficulty in finding isolated atypia in EP and the higher prevalence of type I neoplasia in the general population. Thus, additional research specifically addressing polyps with atypia and type II endometrial neoplasia is necessary and shall be conducted by our group. It is also noticeable that some of our samples were analyzed in TMA format while EP slides were mounted using conventional methods. In order to prevent any bias that could result from staining interference, all slides were prepared in an automated manner to standardize staining. Another point that should be considered is the immunohistochemical scoring system used here. Automation is a reliable alternative for the assessment of immunohistochemical results. However, in this study, immunostaining was assessed visually to avoid misinterpretation due to the highly heterogeneous morphologic quality of the samples. Thus, subjectivity was chosen in favor of reliability, except when samples were inadequate for analysis. Furthermore, the method proposed for the analysis of the expression of claudins and metalloproteinases, which includes different parameters (pattern of membrane staining, staining intensity, and staining %), is highly informative and helps understanding the mechanisms underlying the pathogenesis of endometrial disorders.

## Conclusion

In this study, the malignant potential of EP could not be determined by assessing the immunoexpression of both ER and PR, Ki-67 cell proliferation index, neovascularization network (endoglin – CD105), cellular adhesion molecules (claudins 3 and 4), and extracellular matrix proteins (MMP-2 and -9). No MMP-2 or -9 expression was observed in any endometrial tissue sample. Further studies are necessary for a better understanding of the biomolecular mechanisms underlying endometrial carcinogenesis.

## Supplementary materials

Figure S1 TMA technique.
**Notes:** Recipient block containing 1 mm diameter and 0.2 mm spacing round cores with samples of endometrial cancer and normal endometrium (control). H stained (40×).
**Abbreviations:** H&E, hematoxylin and eosin; TMA, tissue microarray.

Figure S2 Immunohistochemical analysis of ER and PR expression in the glandular epithelium of endometrial polyp and endometrial cancer samples.
**Notes:** (**A**) Immunochemistry showing score 2+ PR nuclear expression in endometrial carcinoma (200×). (**B**) Immunochemistry showing score 4+ PR nuclear expression in endometrial polyp (200×).
**Abbreviations:** ER, estrogen receptor; PR, progesterone receptor.

Figure S3 Immunohistochemical analysis of endothelial marker in the stroma of endometrial polyps and endometrial cancer samples.
**Notes:** (**A**) Immunochemistry showing CD105/endoglin expression in endothelial cells of endometrial cancer newly formed stromal capillaries (200×). (**B**) Immunochemistry showing CD105/endoglin expression in endothelial cells of endometrial polyp (200×).

Figure S4 Immunohistochemical analysis of cell proliferative index Ki-67 nuclei expression in endometrial polyp (200×).

Figure S5 Immunohistochemical analysis expression of claudins 3 and 4 in endometrial polyps and endometrial cancer samples.
**Notes:** (**A**) Immunochemistry showing claudin 3 focal membrane staining and moderate intensity pattern in endometrial cancer cells (400×). (**B**) Immunochemistry showing claudin 4 diffuse membranous pattern and moderate intensity staining in endometrial polyp (400×).

Figure S6 Immunohistochemical analysis showing nearly absence of MMP-2 and -9 expression in all groups.
**Notes:** (**A**) MMP-9 immunohistochemical expression in endometrial cancer (400×). (**B**) MMP-2 immunohistochemical expression in endometrial polyp (200×).

## Figures and Tables

**Table 1:** Clinical, epidemiological, and anthropometric data on the 60 patients diagnosed with either EP (30 women) or EC (30 women)

Variables	EP	EC	*P*-value
Age (years)^a^	52.2 (±10.9)	60.3 (±10.9)	<**0.001**
Parity^b^	3 (0; 8)	2 (0; 6)	≥0.05
BMI (kg/m^2^)^c^	31.9 (±6.1)	30.2 (±4.8)	≥0.05
Menopause^d^	17 (56.7)	27 (90.0)	<**0.05**
Hormone replacement therapy^d^	3 (10.0)	6 (20.0)	≥0.05
Arterial hypertension^d^	17 (56.7)	21 (70.0)	≥0.05
Diabetes mellitus^d^	6 (20.0)	8 (26.7)	≥0.05
Breast cancer^d^	2 (6.7)	2 (6.7)	≥0.05

**Notes:**

a ANOVA followed by the Tukey’s test; mean (±standard deviation).

b Mann–Whitney nonparametric test; mean (minimum; maximum).

c Student’s *t*-test for independent groups; mean (±standard deviation).

d Goodman’s test for associations; Absolute number (percentage). Bold represents statistically significant values.

**Abbreviations:** ANOVA, analysis of variance; BMI, body mass index; EC, endometrial cancer; EP, endometrial polyps.

**Table 2:** Immunohistochemical parameters in patients with EP (30 women), EC (30 women), or normal endometrium (control, 30 women)

Immunohistochemical markers	EP	EC	Control	*P*-value
Estrogen receptor*	2.5+ (1.0+; 4.0+)^a^	1.0+ (0.0; 4.0+)^a^	4.0+ (0.0; 4.0+)^b^	<**0.001**
Progesterone receptor*	3.0+ (0.0; 4.0+)^a,b^	2.0+ (0.0; 4.0+)^a^	4.0+ (0.0; 4.0+)^b^	<**0.05**
CD105*	20.8 (12.0; 48.7)^b^	18.0 (0.0; 45.0)^b^	6.5 (0.0; 26.0)^a^	<**0.001**
Ki-67*	<1.0% (0.0; 15.0%)^b^	2.0% (0.0; 100.0%)^b^	0.0% (0.0; 0.0)^a^	<**0.001**
Claudin 3 (membrane pattern)*	1.0 (0.0; 2.0)^a,b^	2.0 (0.0; 3.0)^b^	1.0 (0.0; 1.0)^a^	<**0.005**
Claudin 3 (intensity)*	3.0 (0.0; 3.0)^b^	3.0 (0.0; 3.0)^b^	2.0 (0.0; 3.0)^a^	<**0.001**
Claudin 3 (stained cells’ counting)*	3.0 (0.0; 4.0)^a^	3.5 (0.0; 4.0)^a,b^	4.0 (0.0; 4.0)^b^	<**0.05**
Claudin 4 (membrane pattern)*	1.0 (0.0; 2.0)	1.0 (0.0; 3.0)	1.0 (0.0; 1.0)	≥0.05
Claudin 4 (intensity)*	2.0 (0.0; 3.0)	2.0 (0.0; 3.0)	2.0 (0.0; 2.0)	≥0.05
Claudin 4 (stained cells’ counting)*	1.0 (0.0; 4.0)	2.0 (0.0; 4.0)	4.0 (0.0; 4.0)	≥0.05
MMP-2 (intensity)*	0.0 (0.0; 0.0)	0.0 (0.0; 0.0)	0.0 (0.0; 0.0)	≥0.05
MMP-2 (stained cells’ counting)*	0.0 (0.0; 0.0)	0.0 (0.0; 0.0)	0.0 (0.0; 0.0)	≥0.05
MMP-9 (intensity)*	0.0 (0.0; 3.0)	0.0 (0.0; 0.0)	0.0 (0.0; 0.0)	≥0.05
MMP-9 (stained cells’ counting)*	0.0 (0.0; 3.0)	0.0 (0.0; 0.0)	0.0 (0.0; 0.0)	≥0.05

**Notes:** Two values (median) followed by the same letter do not differ (*P*>0.05).

* Kruskal–Wallis test with Dunn’s multiple comparison test; median (minimum; maximum). Bold represents statistically significant values.

**Abbreviations:** EC, endometrial cancer; EP, endometrial polyps.
