# Supplementary figures and images for: Immunohistochemical expression of hormone receptors, Ki-67, endoglin (CD105), claudins 3 and 4, MMP-2 and -9 in endometrial polyps and endometrial cancer type I
Source: Onco Targets Ther. 2018 Jul 9;11:3949–58. doi: 10.2147/OTT.S160014 (PMC6042493; doi:10.2147/OTT.S160014)

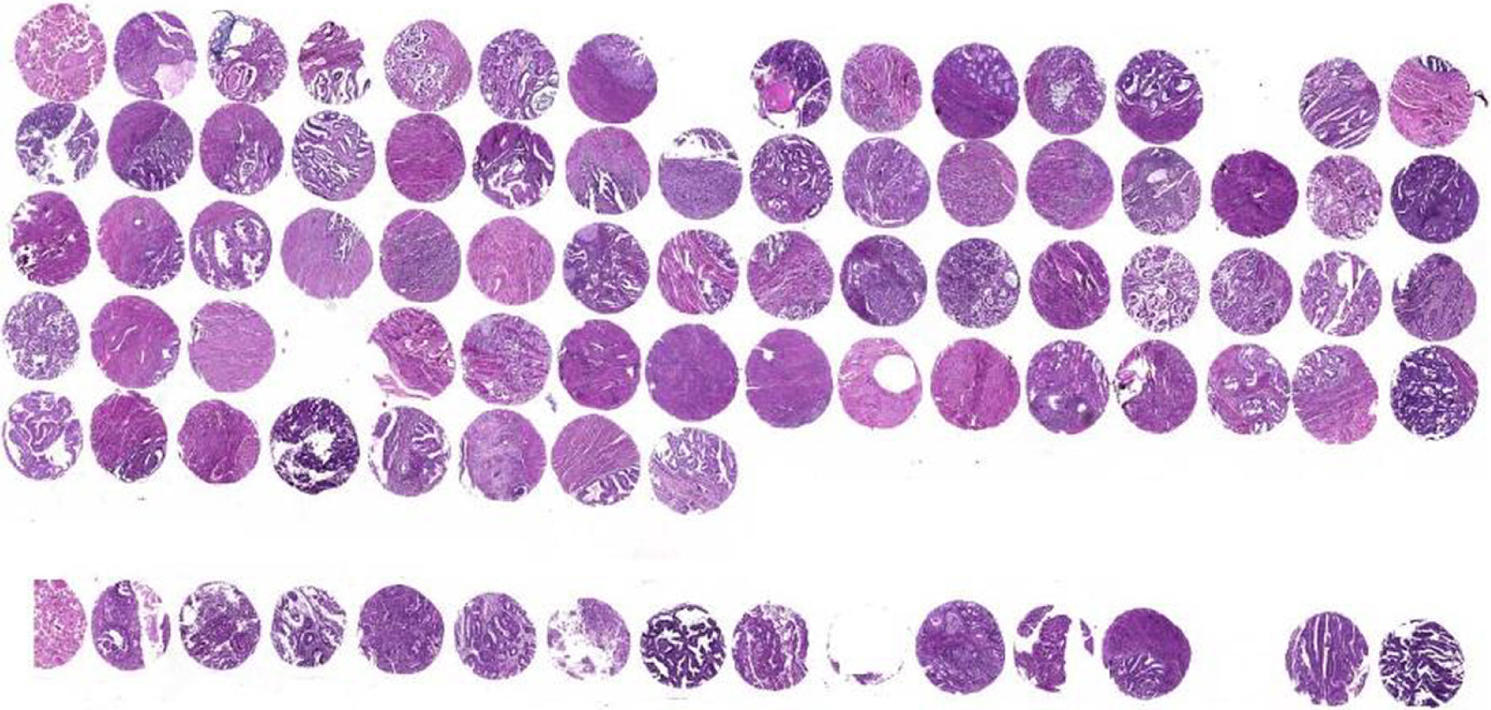

Supplement: Figure S1 — TMA technique. Notes: Recipient block containing 1 mm diameter and 0.2 mm spacing round cores with samples of endometrial cancer and normal endometrium (control). H stained (40×). Abbreviations: H&E, hematoxylin and eosin; TMA, tissue microarray. [file ott-11-3949s1.tif]

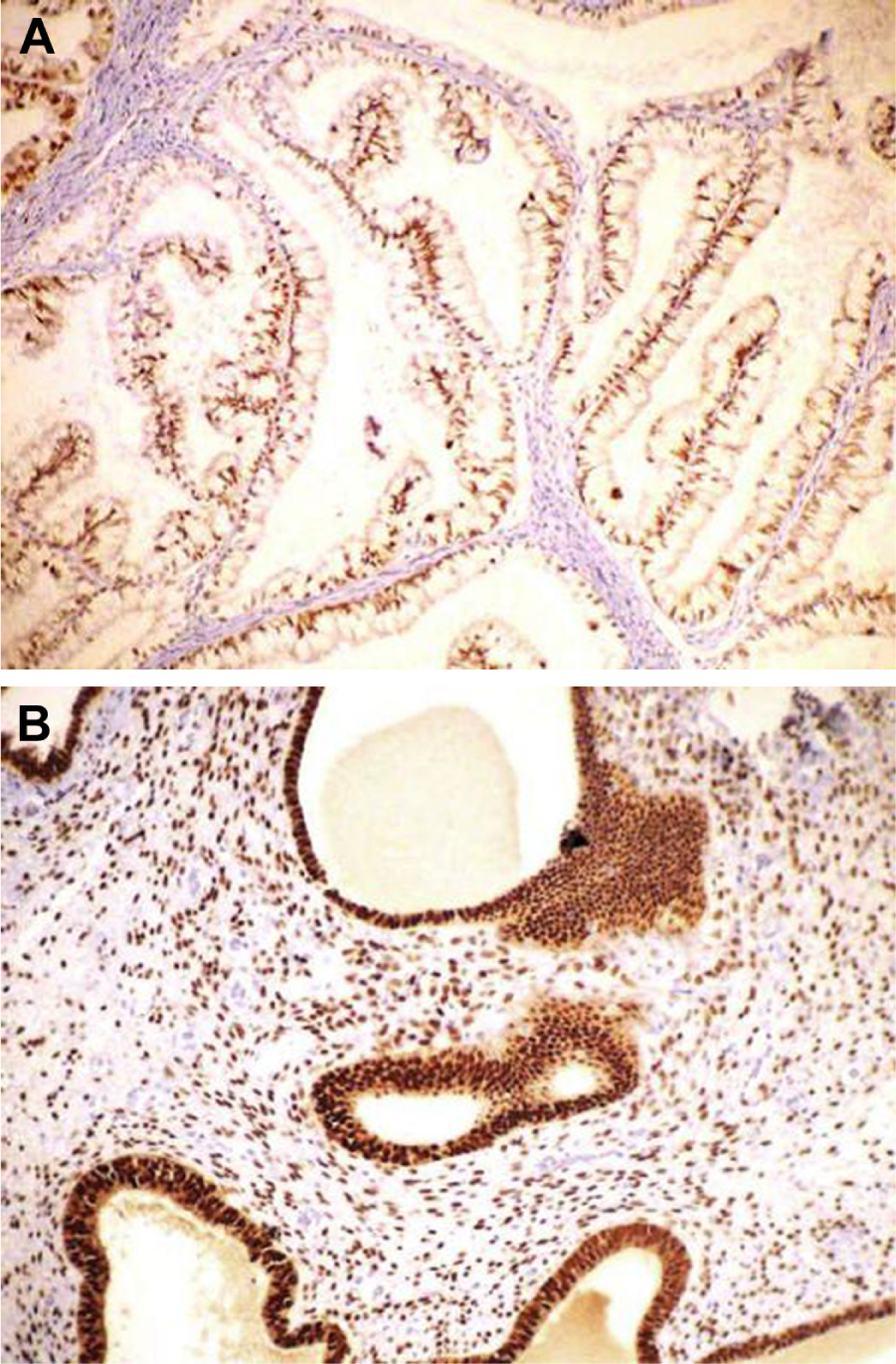

Supplement: Figure S2 — Immunohistochemical analysis of ER and PR expression in the glandular epithelium of endometrial polyp and endometrial cancer samples. Notes: (A) Immunochemistry showing score 2+ PR nuclear expression in endometrial carcinoma (200×). (B) Immunochemistry showing score 4+ PR nuclear expression in endometrial polyp (200×). Abbreviations: ER, estrogen receptor; PR, progesterone receptor. [file ott-11-3949s2.tif]

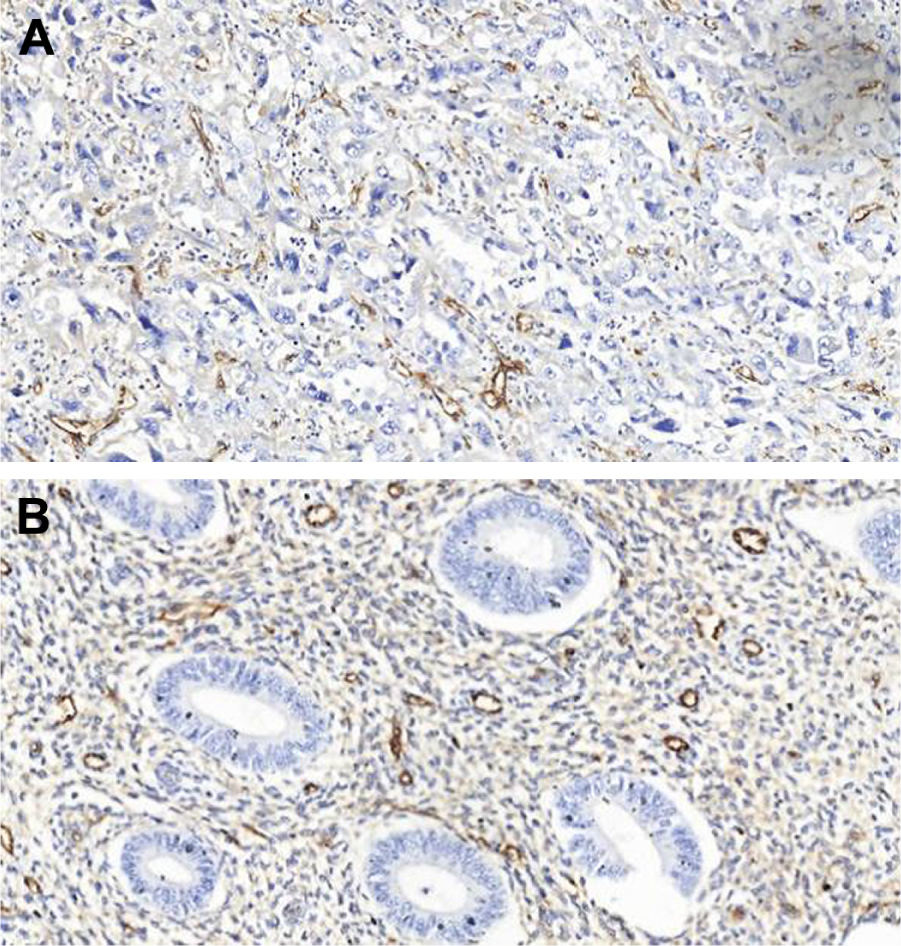

Supplement: Figure S3 — Immunohistochemical analysis of endothelial marker in the stroma of endometrial polyps and endometrial cancer samples. Notes: (A) Immunochemistry showing CD105/endoglin expression in endothelial cells of endometrial cancer newly formed stromal capillaries (200×). (B) Immunochemistry showing CD105/endoglin expression in endothelial cells of endometrial polyp (200×). [file ott-11-3949s3.tif]

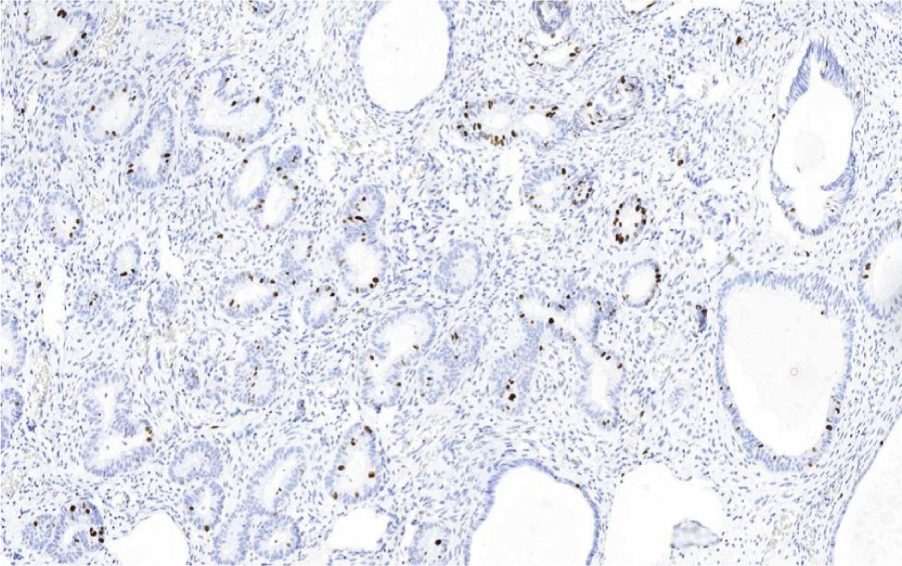

Supplement: Figure S4 — Immunohistochemical analysis of cell proliferative index Ki-67 nuclei expression in endometrial polyp (200×). [file ott-11-3949s4.tif]

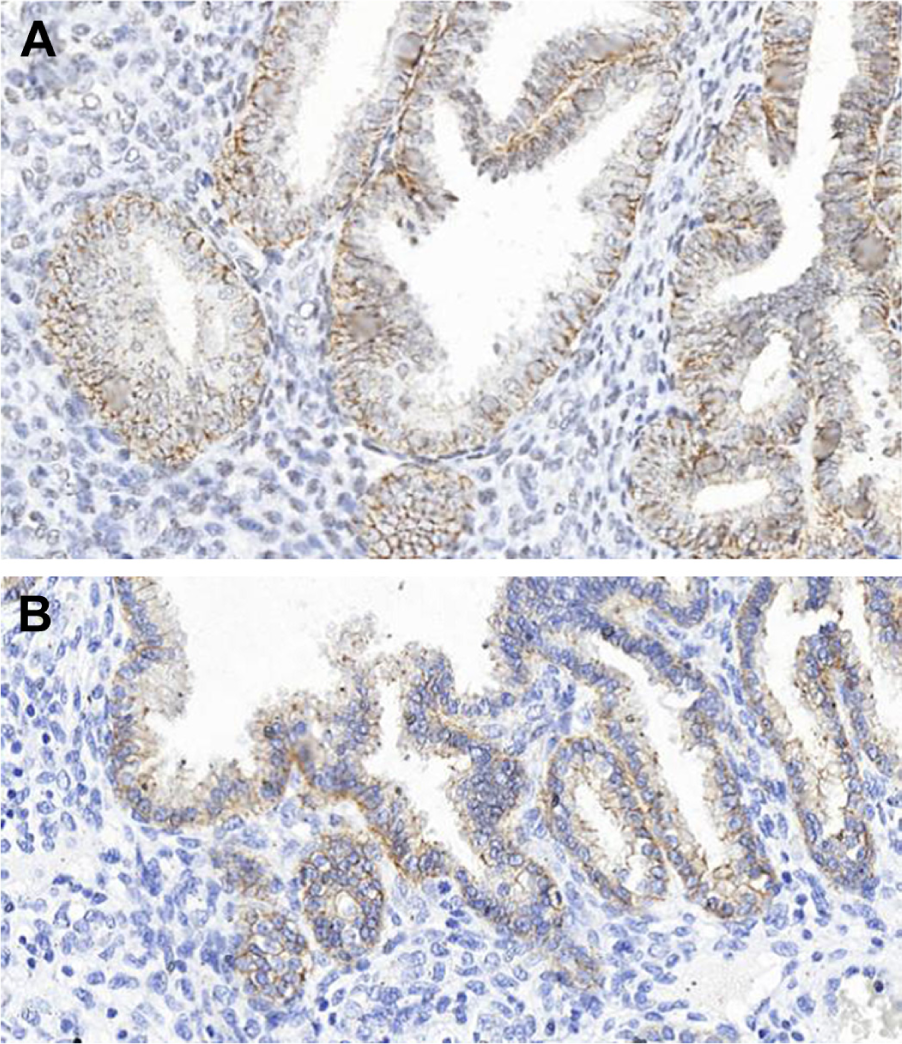

Supplement: Figure S5 — Immunohistochemical analysis expression of claudins 3 and 4 in endometrial polyps and endometrial cancer samples. Notes: (A) Immunochemistry showing claudin 3 focal membrane staining and moderate intensity pattern in endometrial cancer cells (400×). (B) Immunochemistry showing claudin 4 diffuse membranous pattern and moderate intensity staining in endometrial polyp (400×). [file ott-11-3949s5.tif]

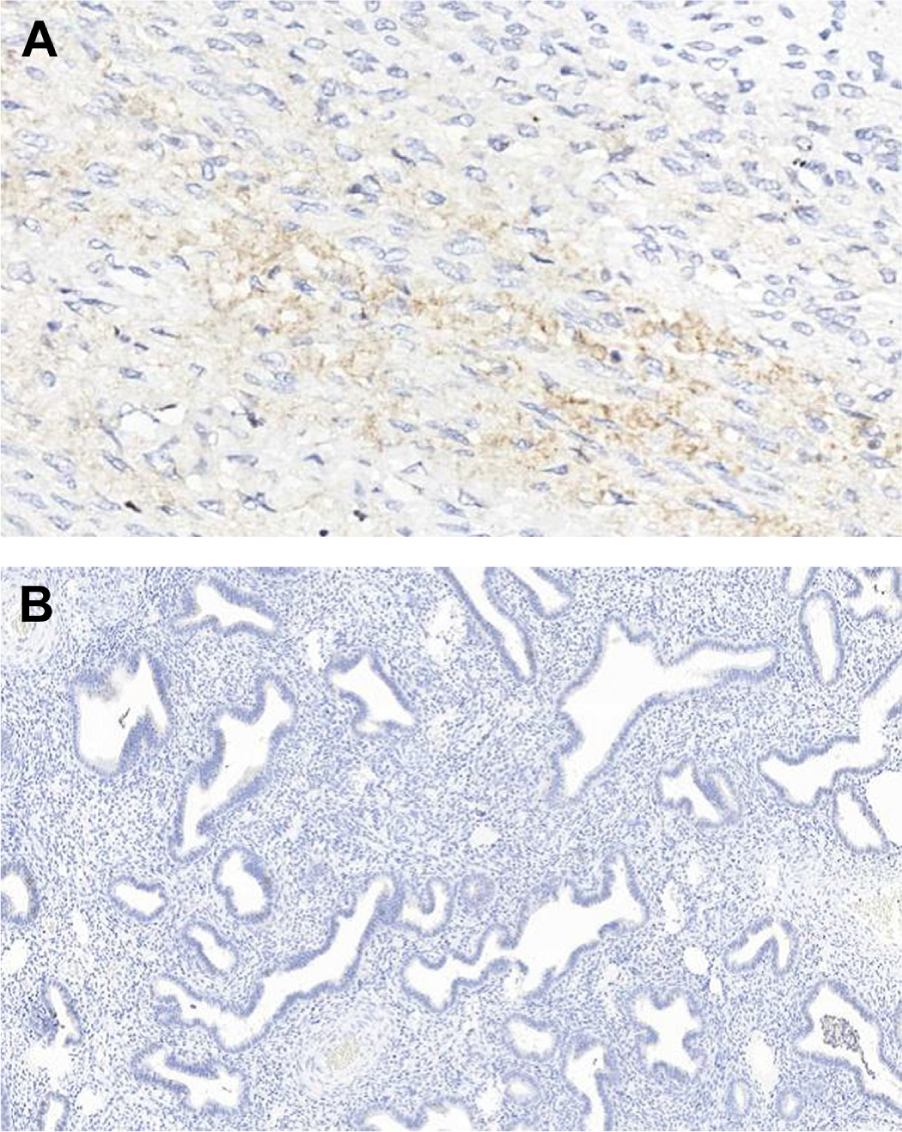

Supplement: Figure S6 — Immunohistochemical analysis showing nearly absence of MMP-2 and -9 expression in all groups. Notes: (A) MMP-9 immunohistochemical expression in endometrial cancer (400×). (B) MMP-2 immunohistochemical expression in endometrial polyp (200×). [file ott-11-3949s6.tif]
